# The Role of Autologous Platelet Concentrates as a Local Antibiotic Delivery System: A Systematic Scoping Review

**DOI:** 10.3390/antibiotics13090856

**Published:** 2024-09-06

**Authors:** Roberta Gasparro, Federica Di Spirito, Maria Domenica Campana, Gilberto Sammartino, Alessandro E. di Lauro

**Affiliations:** 1 Department of Neurosciences, Reproductive Sciences and Oral Sciences, Section of Oral Surgery, University of Naples Federico II, 80131 Naples, Italy; roberta.gasparro@unina.it (R.G.); mariadomenica.campana@unina.it (M.D.C.); alessandroespedito.dilauro@unina.it (A.E.d.L.); 2 Department of Medicine, Surgery and Dentistry, University of Salerno, 84081 Salerno, Italy; fdispirito@unisa.it

**Keywords:** platelet-rich fibrin, platelet-rich plasma, antibiotic, carrier

## Abstract

Objectives: Ongoing research has begun to develop innovative approaches to deliver local antibiotics while minimizing systemic side effects, antimicrobial resistance, and limited tissue penetration. Autologous platelet concentrates (APCs) offer promise in delivering antibiotics directly to infection sites. Despite the interest, a comprehensive evaluation of their effectiveness is lacking. Therefore, this systematic scoping review aims to collect and appraise studies regarding the efficacy of APCs in delivering antibiotics. Methods: A systematic electronic search of PubMed, Scopus, and Web of Science, using a combination of keywords, was conducted up to February 2024. Articles addressing the use of APCs as a local antibiotic delivery system were included. Results: A total of 13 articles, including 10 in vitro studies, 1 in vitro and clinical study, 1 ex vivo study, and 1 clinical study, were selected. Antibiotic loading capacity and release was confirmed in all studies using doxycycline, gentamicin, linezolid, vancomycin, metronidazole, and penicillin. In addition, the antibacterial effect was obtained mainly against *E. coli.*, *P. aeruginosa*, *S. mitis*, *H. influenzae*, *S. pneumoniae*, and *S. aureus*. Conclusions: The incorporation of antibiotics into APCs has been proven to facilitate the effective release of antimicrobial agents at optimal concentrations, potentially reducing the incidence of post-operative infections, substituting, or augmenting systemic antibiotic treatment while retaining APCs’ inherent healing properties.

## 1. Introduction

In the field of modern medicine, the search for effective strategies to combat bacterial infections while minimizing systemic side effects remains a critical effort. Traditional antibiotic therapies, while potent, often encounter challenges such as limited tissue penetration, systemic toxicity, and antimicrobial resistance [1,2]. Consequently, there has been a growing interest in developing innovative approaches to deliver antibiotics directly to the site of infection, thereby maximizing efficacy and minimizing adverse effects [3,4,5]. Various delivery systems for topical antibiotic release, including hydrogels, nanoparticles, and polymers, have undergone testing [6,7,8].

One such approach is using autologous platelet concentrates (APCs) as a local antibiotic delivery system. Platelet concentrates, such as PRF (platelet-rich fibrin), PRP (platelet-rich plasma), PRGF (platelet rich in growth factors), CGF (concentrated growth factor), and i-PRF (injectable platelet-rich fibrin), are currently employed in clinical and surgical settings for tissue regeneration. They offer various potential benefits, such as promoting the regeneration of both hard and soft tissues, aiding in local hemostasis, and accelerating wound healing [9,10]. These attributes make them suitable therapeutic options across different medical fields, such as plastic surgery and dermatology, orthopedics and sport medicine, and veterinary medicine, and are widely applied in periodontology, endodontics, oral surgery, implantology, and oral medicine [11]. Several techniques for platelet concentrates are available in a liquid injectable form or in clot form; therefore, there are various products with different biological features. Briefly, in PRP techniques, the patient’s own blood is collected with anticoagulants and immediately undergoes two centrifugation processes. Finally, the obtained platelet concentrate is activated with thrombin and/or calcium chloride (or similar factors) to trigger platelet activation and fibrin polymerization. For PRF, the blood is collected without any anticoagulant and immediately centrifuged in a single step, which allows for the formation of a PRF clot. This product does not need any activation since no thrombin or calcium chloride is required, making the procedure simpler and easier for clinicians to use. For PRGF preparation, the patient’s own blood is collected with anticoagulants and immediately undergoes one centrifugation process. The fundamental concept involves harvesting and concentrating the most active components from blood samples, including platelets, fibrin, and potentially leukocytes, to prepare them in a form suitable for clinical use. APCs are rich in growth factors and, in addition, possess inherent antimicrobial properties, making them an attractive candidate for enhancing antibiotic therapy [12,13]. Moreover, using a patient’s own blood eliminates the risk of immune rejection and disease transmission, and it reduces potential complications related to foreign materials. With the regenerative potential of platelets and their ability to modulate immune responses, APCs offer a promising tool for promoting the local delivery of antibiotics, thereby potentially overcoming some of the limitations associated with conventional antibiotic treatments.

Scoping reviews are commonly used for “reconnaissance”—to clarify working definitions and conceptual boundaries of a topic or field. Scoping reviews are particularly valuable when the existing literature has not been thoroughly reviewed or when it is vast, complex, or heterogeneous, making a more detailed systematic review impractical [14,15,16]. Three methods of platelet concentrate drug-loading have been described in the literature: by the addition of antibiotics to PRP before coagulation, by the addition of antibiotics to blood before centrifugation (for PRF), and by injection into the PRF membrane after centrifugation [17]. Despite the ongoing interest in APCs as a local antibiotic delivery system, a comprehensive evaluation of their effectiveness based on in vitro and in vivo studies is still lacking. Therefore, this systematic scoping review aims to fill this gap by synthesizing the existing body of literature to provide a global understanding of the efficacy of APCs in delivering antibiotic therapy. In detail, this review aims to evaluate the effectiveness of autologous platelet concentrates (PRF, PRP, PRGF, CGF, and i-PRF) as sole carriers or scaffolds for local antibiotic delivery, focusing on antibiotic loading capacity, release kinetics, and antibacterial effects in both in vitro and in vivo studies.

## 2. Materials and Methods

The protocol for this review was based on the Joanna Briggs Institute’s guidelines for systematic scoping reviews. A research question was formulated, incorporating inclusion criteria for participants, intervention, comparison, outcome, and study design (PICOS), as outlined in Table 1, before initiating the review.

### 2.1. Literature Search

Three electronic databases (PubMed, Scopus, Web of Science) were explored up to 29 February 2024, using combinations of keywords and MeSH terms according to the database rules (Table 2). A manual search of dental journals (*The Journal of the American Dental Association*, *BMC Oral Health*, *Quintessence International*, *Journal of Oral and Maxillofacial Implants*, and the *International Journal of Oral Implantology*) and a further search among the references of the included papers were performed. An effort to explore the gray literature was performed by searching among the conference abstracts published on the Web of Science and Scopus and on the databases of scientific dental congresses (International Association of Dental Research). Two authors (RG and MDC) carried out the electronic literature search separately. Articles addressing the use of autologous platelet concentrates as a local antibiotic delivery system were included. After title and abstract screening, the articles were selected for full-text reading. Whenever differences in the judgment of the eligibility of the title and abstract occurred, full texts were included for final assessment. Dual publications, narrative reviews, updated publications, data from ongoing studies, and drugs other than antibiotics were excluded. Articles written in any language other than English were excluded. Disagreements between the two investigators were resolved through discussion; if needed, a third operator (GS) was contacted for the final decision.

### 2.2. Data Extraction

Data were independently extracted by two authors (RG and MDC) using a pre-determined extraction form. In cases where the information in the articles was unclear, the authors were not contacted for additional details. The following data were extracted: author, publication year, study design, intervention and control groups, outcome measures, results, and author’s conclusion.

## 3. Results

### 3.1. Selection of Studies

**Figure 1:**
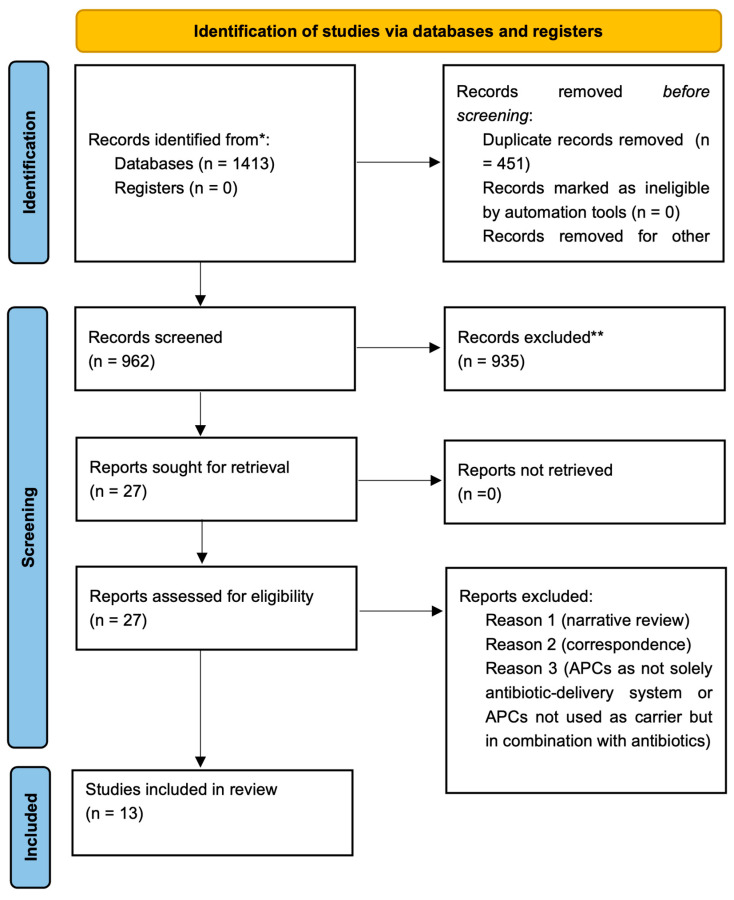
PRISMA flow diagram 2020.

In total, the initial search strategies showed 1413 articles. After duplicate removal, 962 articles remained for title and abstract evaluation. A total of 935 papers were excluded due to a mismatch with inclusion criteria, and 27 articles were retained for final full text review. Finally, 13 articles [17,18,19,20,21,22,23,24,25,26,27,28,29] including 10 in vitro studies [17,18,19,20,22,23,25,26,27,28], 1 ex vivo study [24], 1 clinical study [21], and 1 in vitro and clinical study [29], which have, so far, evaluated the potential of APCs as an antibiotic delivery system, were selected. Reports were excluded for the following reasons: narrative reviews, correspondence, and APCs not used as antibiotic delivery systems (for completion, see Supplementary Table S1: excluded studies and reasons). By excluding these types of reports, the scoping review could ensure a more precise, relevant, and systematic mapping of the literature that directly addresses the specific research objectives [30]. The results of the selected studies were classified under the following subheadings: antibiotic loading capacity of APCs, release kinetics of antibiotics, and antibacterial effects of loaded APCs (in vitro or in vivo). Some studies touched upon different characteristics; therefore, they were included in two/three categories. Furthermore, potential interactions between antibiotics and the structure of APCs, as well as whether APCs modified the characteristics of antibiotics, were reported. The Preferred Reporting Items for Systematic Reviews and Meta-Analyses (PRISMA) flow diagram 2020 in Figure 1 depicts the flow of the included studies through each phase of the review process. The characteristics of the included studies are summarized in Table 3 (for completion, see Supplementary Table S2: summary of the outcomes evaluated in the included studies).

### 3.2. In Vitro Studies

#### 3.2.1. Antibiotic Loading Capacity of APC

Six studies evaluated the antibiotic loading capacity of APC [17,18,20,25,26,28]. In a study by Bennardo et al. [17], an increasing amount of gentamicin, linezolid, and vancomycin was added to L-PRF tubes. Since vancomycin interfered with PRF formation, only the supernatant of gentamicin and linezolid samples was collected and analyzed at different times. The analysis of the supernatant released from PRF indicated that gentamicin and linezolid were effectively bound to the PRF membranes. Dubnika [18] investigated the use of injectable PRF (i-PRF) as a delivery system, incorporating vancomycin hydrochloride (VANKA) via two carrier types—liposomes and microparticles—suspended in PRF before clot formation. Control samples consisted of PRF without VANKA carriers. The findings revealed that VANKA in PRF without a carrier system did not achieve controlled antibiotic loading. In a study by Ercan et al. [20], T-PRF (Titanium-PRF) was used to deliver doxycycline (Doxy) through injection, compared to doxycycline-loaded collagen. T-PRF showed approximately seven times higher doxycycline loading (281 ± 43 mg/g) compared to collagen (47 ± 4 mg/g). Straub’s 2022 study [25] examined the properties of PRF in 24 patients with osteonecrosis of the jaw (ONJ) receiving systemic ampicillin/sulbactam. PRF samples were taken 10 min after intravenous antibiotic administration, revealing that the antibiotic concentrations in PRF (13.52 μg/100 mg for ampicillin and 5.31 μg/100 mg for sulbactam) were comparable to plasma levels (12.05 μg/100 mg for ampicillin and 5.67 μg/100 mg for sulbactam). In a follow-up study by Straub in 2023 [26], 33 patients were administered a single dose of ampicillin/sulbactam (2 g/1 g) with three different PRF preparation protocols (A: 1300 rpm, 8 min; B: 2300 rpm, 12 min; C: 1500 rpm, 14 min). All protocols achieved similar high concentrations of ampicillin/sulbactam in PRF (150 μg/mL), comparable to plasma levels. Finally, Straub et al. [28] in 2024 produced PRF membranes from 36 patients receiving intravenous clindamycin (CLI) and measured CLI concentrations using liquid chromatography with tandem mass spectrometry. The study found that the mean CLI concentration in PRF was 0.7 μg/100 mg, significantly lower than in plasma (*p* < 0.05).

#### 3.2.2. Release Kinetics of Antibiotic

Six studies evaluated drug release kinetics [17,18,19,20,22,29]. In the study of Bennardo et al. [17], an ANOVA test showed the significant impact of antibiotic quantity and time on gentamicin release (*p* < 0.001) and on linezolid release (*p* < 0.001). On the contrary, in the study of Dubnika [18], the introduction of VANKA in PRF scaffolds without a carrier system did not ensure the controlled delivery of the active VANKA form at the therapeutic effect level. Egle and collaborators [19] evaluated the effect of PRF, in three healthy volunteers, as a carrier of clindamycin phosphate (CLP). CLP release from PRF matrices was determined by incubating PRF matrices for 0.25, 0.5, 1, 2, 4, 6, 17, and 24 h, and a burst release of CLP was observed for all PRF_CLP samples in the first incubation hour, when 80% of the encapsulated CLP was released. In the study of Ercan et al. [20], the release of doxycycline was monitored with time by using UV-Vis spectroscopy (PG Instruments T80+) at a wavelength of 350 nm between T-PRF and collagen. As a result, 25% of the loaded doxycycline was released from T-PRF compared to only 12% from the collagen control group over a 72 h period. In the study by Knafl and colleagues [22], the release kinetics of amikacin, teicoplanin, and polyhexanide from a PRF layer were evaluated. The findings indicated that more than 1000 mg/L of teicoplanin was released within the first 24 h, with a concentration of 28.22 mg/L remaining after 168 h. Amikacin release exceeded 10,000 mg/L within the first 24 h and remained at 120.8 mg/L after 120 h. In the study of Rafiee [24], i-PRF loaded with metronidazole, ciprofloxacin, and minocycline showed a burst release within the first 24 h, followed by sustained maintenance of the three antibiotics up to 14 days. The control group (i-PRF without antibiotics) could not sustainably release the antibiotics. Wang and collaborators [29] used PRP as a local antibiotic delivery system for vancomycin (VAN) and ceftazidime (CAZ). The results showed that about 60% of the total VAN and CAZ dose was released within 10 min; then, the release rate gradually decreased. However, 90% of clindamycin was released within 10 min. Interestingly, an amount 10 times over the minimum inhibitory concentration was presented after 72 h.

#### 3.2.3. Antibacterial Effects of Loaded APCs

All the in vitro studies included in this scoping review evaluated the antimicrobial effect of APC-loaded antibiotics [17,18,19,20,22,23,24,25,26,27,28,29,30,31,32,33,34,35,36,37,38,39]. Bennardo et al. [17] evaluated PRF membranes loaded with gentamicin and linezolid against strains of *E. coli*, *P. aeruginosa*, *S. mitis*, *H. influenzae*, *S. pneumoniae*, and *S. aureus*, comparing them to control PRF without antibiotics at 24, 48, 72, and 96 h. Gentamicin-PRF exhibited strong antibacterial activity against all tested microorganisms, while linezolid-PRF showed similar efficacy, except against *E. coli* and *P. aeruginosa*, where its activity was comparable to the control. In Dubnika’s study [18], the introduction of vancomycin (VANKA) into PRF scaffolds with a carrier system demonstrated complete antibacterial activity against *S. aureus* for 48 h, although the effectiveness declined rapidly after the first 24 h. Egle [19] found that clindamycin phosphate (CLP) incorporated into PRF showed significantly lower minimal bactericidal concentrations against *S. aureus* and *S. epidermidis*, compared to a pure CLP solution, after 18 h of incubation at 37 °C. Ercan [20] evaluated the antibacterial effects of doxycycline-loaded T-PRF against *S. aureus* and *P. aeruginosa*, comparing it to doxycycline-loaded collagen over various incubation times up to 7 days. T-PRF/Doxy showed the largest inhibition zones (32 ± 6 mm for *P. aeruginosa* and 37 ± 5 mm for *S. aureus*), while T-PRF alone had smaller inhibition zones (10 ± 5 mm), and the collagen/Doxy group showed no inhibition. Knafl et al. [22] demonstrated that teicoplanin and amikacin released from PRF were effective against methicillin-sensitive and methicillin-resistant *Staphylococcus aureus*, *Pseudomonas aeruginosa*, *Klebsiella pneumoniae*, and *K. pneumoniae 4MRGN* for nearly a week. However, polyhexanide showed antimicrobial activity only for the first 24 h. Polak [23] prepared PRF with metronidazole, clindamycin, and penicillin before centrifugation, using collagen sponges as controls. The antibiogram assay revealed minor antibacterial activity for PRF with saline, while all antibiotic-loaded PRFs exhibited significant antibacterial effects against *S. aureus* and *Fusobacterium nucleatum.*

Rafiee et al. [24] evaluated an injectable platelet-rich fibrin (i-PRF) scaffold with a triple antibiotic mixture (ciprofloxacin, metronidazole, and minocycline) against *Actinomyces naeslundii* and *Enterococcus faecalis* biofilms in an infected root canal model. The i-PRF with antibiotics showed the highest antibacterial activity against *A. naeslundii* but similar effectiveness to the control group against *E. faecalis*, reducing live bacteria counts by up to 92%. Straub et al. [25] assessed the antimicrobial properties of PRF loaded with ampicillin/sulbactam against *Haemophilus influenzae*, *Streptococcus pneumoniae*, *S. aureus*, and *Escherichia coli* after 20 h. The inhibition zones of PRF were comparable to standard ampicillin/sulbactam discs, while control PRF from patients without antibiotic therapy showed no inhibition. In a follow-up study by Straub in 2023 [26], PRF was prepared using three different protocols and evaluated for its antimicrobial properties against the same bacteria. Protocol B resulted in the largest inhibition zones. Straub [27] also investigated PRF’s antimicrobial properties with amoxicillin/clavulanic acid and ampicillin/sulbactam in different doses and storage conditions. Fresh PRF and single oral doses of amoxicillin showed varying inhibition zones, with significant differences observed between parenteral and oral applications.

In 2024, Straub et al. [28] examined clindamycin-loaded PRF for its antimicrobial effects against several bacteria. Fresh PRF exhibited mean inhibition zones of 17.3 mm for *S. aureus* and 25.8 mm for *Fusobacterium nucleatum*, while stored PRF had slightly larger zones. Wang et al. [29] used PRP as a local antibiotic delivery system for vancomycin and ceftazidime, finding that the inhibition zones were like those of the antibiotics alone.

#### 3.2.4. Mutual Interaction: Antibiotics’ Impact on APC Structures and Vice Versa

Five studies reported a mutual interaction between antibiotics and APC properties [17,19,20,23,29]. In the study of Bennardo [17], vancomycin interfered with PRF formation; therefore, this sample was eliminated by the study before the evaluation of antibiotic release and antimicrobial effect. Egle and collaborators [19] reported that the use of PRF as a carrier of clindamycin phosphate ensured that the structural changes in CLP occurred as a more active form of clindamycin. Ercan [20] showed that the potential fibrinophilic properties of Doxy seemed to strengthen the structure of T-PRF. In the study of Polak [23], the addition of antibiotic solutions at volumes of 2 or 1 mL led to significant changes in PRF’s physical properties, while the addition of a 0.5 mL solution did not. Wang [29] showed that adjusting the dosage of vancomycin and ceftazidime in the PRP delivery system led to a reduction in growth factor concentration released by PRP and caused disruption in the structure of platelet-rich fibrin beams and the fibrin network in a manner dependent on the dosage. In detail, high doses of vancomycin inhibited fibrin formation, with low doses (1 mg) resulting in more fibrin but with a disordered and nonhomogeneous structure. Clindamycin at 100 mg completely inhibited fibrin formation, leaving only erythrocytes visible under fluorescence microscopy. By contrast, 1 mg and 10 mg doses of clindamycin produced a homogeneous and prominent fibrin network. For ceftazidime, both 1 mg and 10 mg doses resulted in a dense and well-organized fibrin structure.

### 3.3. In Vivo Studies

In vivo studies did not evaluate the antibiotic loading capacity of APCs nor the release kinetics of antibiotics nor the antibacterial effects of loaded APCs, but they analyzed the clinical effect of loaded APCs. Of all the included studies, one was a clinical case report [21], and another one was an in vitro and case report [29]. Kadam and colleagues [21] conducted an in vivo study to assess the clinical and radiographic effectiveness of amoxicillin-incorporated PRF in managing periodontal intrabony defects. Sixteen defects were treated with either amoxicillin-incorporated PRF (test site) or PRF alone (control site). Clinical parameters, including probing pocket depth (PPD), relative attachment level (RAL), and relative gingival margin level (RGML), were measured at baseline and 6 months post-operatively. The wound healing index (WHI) was evaluated 7 and 14 days post-surgery. Defect fill was assessed at baseline and at 6 months using cone beam computed tomography (CBCT). A comparative analysis of mean PPD, RAL, and RGML between the groups revealed no statistically significant differences. However, the WHI showed significantly improved healing in the test group 7 days post-operatively. There was no significant difference in defect fill changes between the control and test groups from baseline to 6 months. In addition to an in vitro study, Wang and collaborators [29] used platelet-rich plasma loaded with antibiotics to treat a case of bone infection by eliminating bacteria and promoting bone healing, which was successfully obtained at the 15-month follow-up.

## 4. Discussion

APCs are well-known products that promote accelerated healing in both soft and hard tissues, and they are widely applied in periodontology, endodontics, oral surgery, implantology, and oral medicine [31,32]. In 2018, Miron and Zhang first proposed the use of APCs as a drug delivery system and proposed their combination with various molecules, including antibiotics [33]. Considering the still-emerging field, the developing literature, and the broad and exploratory research question, we decided to conduct a scoping review rather than a systematic review based on the current state of the literature and the research objectives [30]. Therefore, this systematic scoping review aimed to synthesize the existing literature on the efficacy of autologous platelet concentrates (APCs) in topical antibiotic delivery.

Articles addressing the use of autologous platelet concentrates as local antibiotic delivery systems were included, generally showing the high antibiotic loading capacity of APCs, slow-release kinetics of the antibiotic, and strong antibacterial effect of loaded APCs, both in in vitro and in vivo studies. When antibiotics are loaded before APC coagulation, they can inhibit the formation of blood clots, making the procedure not applicable. In the study of Bennardo [17], vancomycin interfered with PRF formation, and this finding was confirmed by Dubnika, where the introduction of VANKA in PRF scaffolds without a carrier system did not ensure the controlled loading capacity of the antibiotic, and there was no specific binding of VANKA to the PRF scaffold [18]. On the contrary, the addition of clindamycin or doxycycline seems to strengthen the properties of PRF clot. This point is crucial because the goal of these systems is not only to guarantee antibiotic release but also to maintain the APC’s ability to deliver growth factors and cytokines, which are essential for enhancing the natural antibacterial effect of APCs [34,35], and this also depends on the fibrin structure of the clot [36]. This effect is ensured by the complement-binding proteins in platelet α-granules, the direct engagement of platelets with microbes, and their participation in antibody-dependent cell cytotoxicity [37]. Meanwhile, white blood cells directly kill bacteria, produce myeloperoxidase, activate the antioxidant-responsive element, and initiate an antigen-specific immune response [38]. Moreover, the choice of anticoagulant in the tubes can influence the resulting fibrin formation. This concern is relevant only for platelet-rich plasma (PRP), which typically uses citrate as the anticoagulant, whereas platelet-rich fibrin (PRF) is prepared without any anticoagulants [39].

When antibiotics are first administered intravenously and blood is collected, the capacity of APC loading can be measured in comparison to plasma concentration. In this case, it is crucial to collect blood samples at the appropriate time to reach clinically relevant concentrations in APCs. Typically, this involves sampling around the expected peak plasma concentration of the antibiotic [40]. For many intravenously administered antibiotics, this may occur towards the end of the infusion or shortly thereafter [41]. This emphasizes the importance of understanding the specific pharmacokinetics of antibiotics to achieve APCs enriched with antibiotics at a therapeutic level of efficacy, considering that age, the route of administration, and body clearance capacity may influence plasma and, consequently, APC drug concentration [42,43]. Ercan et al. [20] reported higher loading and subsequent release of doxycycline from T-PRF compared to collagen, indicating that T-PRF might be a more efficient carrier for certain antibiotics. Moreover, both Egle et al. [19] and Wang et al. [29] observed a rapid burst release of antibiotics within the initial phase, which may be crucial for achieving immediate therapeutic levels. Specifically, Egle et al. [19] noted an 80% release of clindamycin phosphate within the first hour, whereas Wang et al. [29] reported that 60% of vancomycin and ceftazidime were released within the first 10 min. Notably, the average maximum duration of antibiotic release was observed in the first 24 h, with the concentration decreasing as a function of antibiotic amount and time. However, a controlled and sustained release of antibiotics over time should be considered for optimal drug delivery in order to prevent the recurrence of infection and reduce the need for repeated antibiotic administration. Indeed, while Bennardo et al. [17] achieved significant release over time for gentamicin and linezolid, Dubnika’s study [18] emphasized the necessity of a carrier system to ensure the controlled delivery of vancomycin. Rafiee et al. [24] observed a burst release within 24 h, followed by sustained release up to 14 days, which is beneficial for prolonged therapeutic effects. Similarly, Knafl et al. [22] demonstrated the sustained release of amikacin and teicoplanin over several days, although polyhexanide was only released for 24 h. The studies overall illustrated the broad-spectrum antibacterial effect of various antibiotics when loaded into APCs. In detail, Bennardo et al. [17] and Dubnika [18] highlighted the efficacy of antibiotics such as gentamicin and vancomycin against multiple bacterial strains. Bennardo et al. [17] showed significant activity against *E. coli*, *P. aeruginosa*, *S. mitis*, *H. influenzae*, *S. pneumoniae*, and *S. aureus*. However, Dubnika [18] found a rapid decline in efficacy after 24 h, highlighting the need for sustained release mechanisms, as already specified. Egle et al. [19] and Polak [23] demonstrated improved efficacy when antibiotics were combined with PRF, showing a significant reduction in the minimum bactericidal concentration and significant antibacterial activity, respectively. Egle et al. [19] reported efficacy against *S. aureus* and *S. epidermidis*. Polak et al. [23] observed significant activity against *S.aureus* and *Fusobacterium nucleatum.* Ercan et al. [20] further emphasized this by showing that doxycycline-loaded T-PRF outperformed collagen-loaded doxycycline in terms of inhibition zone diameter, particularly against *S. aureus* and *P. aeruginosa*. Knafl et al. [22] and Rafiee et al. [24] provided insights into sustained release and prolonged antibacterial activity, with Knafl et al. [22] noting an almost week-long effect for certain antibiotics, particularly against methicillin-susceptible *Staphylococcus aureus*, methicillin-resistant *Staphylococcus aureus*, *Pseudomonas aeruginosa*, and *Klebsiella pneumoniae.* Rafiee et al. [24] observed significant antibacterial activity against *Actinomyces naeslundii* and *Enterococcus faecalis*. By contrast, Wang et al. [29] confirmed that PRP does not impair the efficacy of antibiotics such as vancomycin and ceftazidime against *S. aureus*, *E. coli*, and *P. aeruginosa*. Finally, the series of studies by Straub [25,26,27,28] consistently showed that PRF loaded with various antibiotics has significant antimicrobial properties, with specific preparation protocols enhancing efficacy against *Haemophilus influenzae*, *Streptococcus pneumoniae*, *Staphylococcus aureus*, *Escherichia coli*, and *Porphyromonas gingivalis*.

### 4.1. Clinical Relevance

APCs, as a novel local antibiotic delivery system, hold several clinically relevant implications. First of all, APCs can deliver high concentrations of antibiotics directly to the site of infection, potentially increasing the efficacy of treatment while limiting systemic side effects and reducing the risk of antimicrobial resistance [44,45] through optimized control of the site, time, and rate of antibiotic release [46,47]. This localized delivery can be particularly beneficial in medical and dental applications where infections are localized and often refractory to systemic antibiotic therapy. Coherently, the use of local antibiotics in the treatment of infected diabetic wounds offers several notable advantages, ensuring high drug concentrations directly at the wound site. This is particularly important in diabetic patients, who often suffer from poor peripheral circulation, hindering the efficacy of systemically administered antibiotics [48]. In complex refractory cases and sites, periodontitis treatment can benefit from topical, beyond systemic, antimicrobial administration. Accordingly, new local antibiotic delivery systems have been developed, providing both immediate and sustained antibacterial effects and improving clinical outcomes in periodontitis treatment [49]. Indeed, various drug delivery system forms, such as gels and fibers, loaded with antibiotics like doxycycline, metronidazole, and minocycline, demonstrated sustained release and effective antibacterial properties in reducing periodontal pathogens [50]. Moreover, tunable systems with doxycycline and lipoxin loaded into biodegradable polymers like polylactic-co-glycolic acid (PLGA) microspheres within a polyisocyanopeptide (PIC) hydrogel showed doxycycline release from 1 to 6 weeks and lipoxin up to 10 days, maintaining significant antibacterial activity against Porphyromonas gingivalis [51]. Similarly, adjunctive antiseptic agents or local antibiotic treatments can improve clinical parameters in patients affected by peri-implantitis treated with gels, microspheres, fibers, and ointments delivering antibiotics like minocycline, doxycycline, lincomycin, erythromycin, and metronidazole directly to peri-implant tissues, thus enhancing local drug concentration [52]. Clinical studies showed significant antibacterial effects, with drug delivery systems loaded with minocycline and doxycycline improving peri-implant clinical outcomes when combined with nonsurgical mechanical debridement [53]. Minocycline and metronidazole gels have also shown substantial reductions in peri-implant mucositis and inflammation, supporting their efficacy [54,55].

Most importantly, the use of local antibiotic delivery may be elective in patients affected by Medication-related Osteonecrosis of the Jaws (MRONJ), because, due to the limited blood supply in necrotic bone tissue, the penetration of systemic antibiotics to the affected areas is hindered, making it difficult for the antibiotics to reach the therapeutic concentrations necessary to combat infection effectively [56,57]. Conversely, the antibacterial effects of locally delivered antibiotics in MRONJ cases has been reported in clinical studies. Minocycline orabase paste has resulted in pain relief and complete healing in several patients, and active oxygen gel has shown promising results in reducing bacterial load and enhancing wound healing through improved oxygenation [58,59]. In particular, minocycline and doxycycline gels ensure that a significant amount of the antibiotic is delivered directly to the affected area and that the drug is released slowly, providing prolonged sustained local drug levels and antibacterial effects [57]. Similarly, active oxygen gel (blue^®^m) has been used to enhance tissue oxygenation and promote wound healing through sustained release [60]. Based on these findings, the use of APCs as a local antibiotic delivery system may positively influence the reduction in infection and hard and soft tissue healing synergically in MRONJ cases. Furthermore, for preventing or treating surgical site infections, antibiotic-loaded gels, fibers, and microspheres deliver high concentrations of antibiotics such as amoxicillin, metronidazole, and doxycycline directly to the surgical site [59]. These systems ensure sustained release over days to weeks, maintaining effective antibiotic levels and reducing infection risk. Clinical studies show that considering both antibiotic- and antiseptic-loaded delivery systems, including amoxicillin/clavulanic acid and chlorhexidine gels, effectively lowered the rates of surgical site infections and complications like dry socket, compared to placebo [59]. Advanced-PRF was recently tested in dry socket disease with success because it significantly reduced pain levels and enhanced the wound-healing process [61]. However, probably, adding antibiotic solutions in PRF in the management of this extraction complication may improve the outcomes.

### 4.2. Limits, Strengths, and Future Perspectives

While this scoping review is comprehensive, it lacks the in-depth analysis typical of systematic reviews because it did not critically evaluate the quality of the included studies. This can be a limitation when detailed evidence synthesis is needed. However, to the best of our knowledge, this current review is the first scoping systematic review on this topic. Future research should prioritize optimizing methods for loading antibiotics into APCs, exploring various antibiotics and their interactions with APCs to ensure effective drug loading and sustained release while maintaining the biological properties of the APCs. A critical gap in current research on the efficacy of autologous platelet concentrates (APCs) to provide appropriate antibiotic system release is the lack of extensive clinical validation. Despite promising preclinical findings, there is a significant need to transition from laboratory-based studies to rigorous clinical trials. In this regard, large-scale clinical trials are needed to validate the efficacy and safety of APC-based antibiotic delivery systems and the standardization of preparation protocols and dosages, which is crucial for widespread clinical use. Innovations in preparation and delivery technology, like advanced centrifugation techniques (e.g., ultracentrifugation, density gradient centrifugation) and co-delivery systems, could enhance the efficacy of APCs by optimizing mutual interaction with the loaded antibiotic. While this review evaluates the most used antibiotic classes in dentistry (amoxicillin, amoxicillin/clavulanic acid, ampicillin), exploring alternative or additional antibiotics such as macrolides could be beneficial for managing resistant infections and more complex cases. Moreover, co-delivery systems may be useful to simultaneously deliver multiple therapeutic agents to a targeted site, enhancing their combined efficacy and optimizing therapeutic outcomes. In conclusion, integrating APCs as a local antibiotic delivery system shows great promise, and ongoing research and clinical validation are essential to fully realize their potential and establish new standards in infection management and tissue regeneration.

## 5. Conclusions

In vitro and in vivo studies investigating the use of autologous platelet concentrates as local antibiotic delivery systems, considered in this scoping review, generally revealed the high antibiotic-loading capacity of APCs, slow-release kinetics of the antibiotic, and the strong antibacterial effect of antibiotic-loaded APCs. APCs as a novel local antibiotic delivery system holds several clinically relevant implications. First, APCs can deliver high concentrations of antibiotics directly to the site of infection, potentially increasing the efficacy of treatment while limiting systemic side effects and reducing the risk of antimicrobial resistance through optimized control of the site, time, and rate of antibiotic release. This approach potentially replaces or supplements systemic antibiotics while preserving the inherent healing properties of APCs. Further in vitro and clinical studies are necessary to both develop high-performance methods for the local delivery of antibiotics using APCs as bio-carriers and to test them for applications in dentistry.

## Figures and Tables

**Table 1:** Eligibility criteria for this present systematic scoping review.

Domain	Inclusion Criteria	Exclusion Criteria
Population	Antibiotics	Drugs other than antibiotics
Intervention	Using autologous platelet concentrates (PRF, PRP, PRGF, CGF, i-PRF) as a sole carrier or scaffold for drug delivery	Using other carriers or scaffolds for drug delivery
Comparison	No antibiotic delivery	
Outcomes	Antibiotic loading capacity of APCs, release kinetics of antibiotic, and antibacterial effects of loaded APCs	
Study Design	In vitro studies, in vivo studies, animal studies, non-comparative studies, case reports, case series, and prospective/retrospective clinical trials	Narrative reviews, systematic reviews with or without meta-analysis, letters to the editors, and short communications

**Table 2:** Search strategy.

Database	Search Strategy	Hits
Pubmed	(“platelet-rich fibrin” [MeSH Terms] OR “platelet-rich plasma” [MeSH Terms] OR “autologous platelet concentrates” [All Fields] OR “platelet-rich in growth factors” [All Fields] OR “PRP” [All Fields] OR “PRF” [All Fields]) AND (“carrier” [Title/Abstract] OR “bio-carrier” [Title/Abstract] OR “delivery” [Title/Abstract]) OR “slow-release” [Title]) AND ((“antibiotic *”) [Title/Abstract] OR “drugs” [Title/Abstract] OR “antimicrobials”) [Title/Abstract])	242
Scopus	TITLE-ABS-KEY (platelet-rich *) OR TITLE-ABS-KEY (autologous AND platelet AND concentrates) OR TITLE-ABS-KEY (prp) OR TITLE-ABS-KEY (prf) AND TITLE-ABS-KEY (carrier) OR TITLE-ABS-KEY (bio-carrier) OR TITLE-ABS-KEY (delivery) OR TITLE-ABS (slow-release) AND (TITLE-ABS-KEY (antibiotic *) OR TITLE-ABS-KEY (drugs) OR TITLE-ABS-KEY (antimicrobials))	730
Web of science	(TS = (autologous platelet concentrates) OR TS = (Platelet-rich *) OR TS = (PRP) OR TS = (PRF)) AND (TS = (* carrier) OR TS = (delivery) OR TS = (slow-release)) AND (TS = (antibiotic *) OR TS = (drug *) OR TS = (antimicrobials))	476

**Table 3:** Characterized study.

Author, Year of Publication	Setting	Study Design	Intervention	Control	Outcome	Results	Conclusion
Bennardo F et al., 2023 [17]	Magna Graecia University of Catanzaro, Italy	In vitro study	L-PRF loaded with gentamicin, linezolid, and vancomycin	L-PRF without antibiotics	Antibiotic release kinetics and antibacterial effects	Gentamicin and linezolid were significantly trapped or bound to the PRF membranes and released over time. Gentamicin-PRF had significant antibacterial activity *against E. coli*, *P. aeruginosa*, *S. mitis*, *H. influenzae*, *S. pneumoniae*, and *S. aureus.* Linezolid-PRF had a comparable activity against *E. coli* and *P. aeruginosa* to the control PRF.	Using PRF loaded with antibiotics after oral surgery may reduce the risk of post-operative infection and replace or enhance systemic antibiotic therapy while preserving the healing properties of PRF.
Dubnika A et al., 2021 [18]	Riga Stradins University	In vitro study	i-PRF loaded with Vancomycin hydrochloride (VANKA) carriers as liposomes and microcapsules	i-PRF without carriers	Antibiotic loading capacity, antibiotic release kinetics, and antibacterial effects	VANKA included in a PRF scaffold without a carrier did not ensure the controlled loading capacity and release of antibiotics. A complete antibacterial effect against *S. aureus* lasted for 48 h, but with a rapid drop of effectiveness after the first 24 h.	This study confirms that the use of a carrier system can ensure controlled VANKA release from PRF for 6 to 10 days.
Egle K et al., 2022 [19]	Riga Stradins University	In vitro study	PRF loaded with clindamycin phosphate (CLP)	PRF without CLP and pure CLP solutions	Antibiotic release kinetics and antibacterial effects	A burst release of CLP was observed for all samples at 0.25, 0.5, 1, 2, 4, 6, 17, and 24 h; at 24 h, 80% was released. A significant decrease in MIC against *S. aureus* and *S. epidermidis* was observed compared to pure CLP solutions.	This modified PRF could be used as a novel method to increase drug delivery and efficacy and to reduce the risk of postoperative infection.
Ercan E. et al., 2022 [20]	Canakkale OnSekiz Mart University	In vitro study	T-PRF loaded with doxyciclin	Collagen combined with doxyciclin	Antibiotic loading capacity, antibiotic release kinetics, and antibacterial effects	In comparison with collagen, approximately sevenfold more Doxy was loaded into T-PRF (281 ± 43 mg/g vs. 47 ± 4 mg/g). A total of 25% of the loaded Doxy was released from T-PRF compared to only 12% from collagen within 72 h. The largest IZD was observed for T-PRF/Dox with 32 + 6 mm and 37 ± 5 mm for *P. aeruginosa* and *S. aureus*, respectively.	T-PRF was shown to have potential autogenous long-term drug-carrying capability for doxycycline.
Kadam S. et al., 2023 [21]	Department of Periodontology, Dr. D. Y. Patil Dental College & Hospital, Pimpri, Pune	In vivo study: prospective randomized controlled trial (RCT)	PRF loaded with amoxicillin	PRF without antibiotic	Clinical and radiographic parameters: PPD, RAL and RGML, WHI and defect fill	Intergroup comparison of mean PPD, RAL, and RGML parameters at baseline and 6 months postoperatively showed no statistically significant difference. Comparison of WHI between groups showed significant healing at 7 days post operatively. Defect fill change showed no significant difference between groups from baseline to 6 months.	Both treatment modalities are equally effective in the treatment of intrabony defects, but the use of PRF/amoxicillin significantly benefited the initial wound healing.
Knafl D. et al., 2017 [22]	Medical University of Vienna	In vitro study	PRF mixed with teicoplanin, amikacin, or polyhexanide	PRF without antibiotics	Antibiotic release kinetics and antibacterial effects	More than 1000 mg/L teicoplanin were released within the first 24 h and 28.22 mg/L after 168 h. Amikacin release was above 10,000 mg/L within the first 24 h and still 120.8 mg/L after 120 h. A release of polyhexanide could be verified for the first 24 h only. Consequently, teicoplanin and amikacin released from PRF showed antimicrobial in vitro effects for almost a week, whereas the antimicrobial effect of polyhexanide could only be verified for the first 24 h.	Wound bandages in wounds treated with PRF plus amikacin or PRF plus teicoplanin can be left for at least five days, regarding antimicrobial efficacy.
Polak D. et al., 2019 [23]	Department of Periodontology, The Hebrew University-Hadassah Medical Center, Jerusalem, Israel	In vitro study	PRF loaded with metronidazole, clindamycin, and penicillin	Collagen sponges with and without antibiotics	Antibacterial effects	PRF with saline had minor antibacterial activity, while all PRFs with antibiotics showed significant antibacterial activity against *S. aureus* or *Fusobacterium nucleatum*.	Platelet-rich fibrin incorporated with antibiotics may be used to reduce the risk of post-operative infection in addition to the beneficial healing properties of PRF.
Rafiee A. et al., 2021 [24]	Shiraz University of Medical Sciences, Shiraz, Iran	In vitro study	i-PRF loaded with metronidazole, ciprofloxacin, and minocycline	i-PRF without antibiotics	Antibiotic release kinetics and antibacterial effects	The test group showed burst release within the first 24 h followed by sustained maintenance of all three antibiotics up to 14 days. The control group could not sustainably release the antibiotics. The highest antibacterial activity against *A. naeslundii* belonged to the group of i-PRF-loaded antibiotics. However, the test and control groups had similar antibacterial properties against *E. faecalis*.	Taken together, the fabricated scaffold could dramatically reduce both total bacterial gene quantification and the number of live bacteria inside the root canal.
Straub A. et al., 2022 [25]	University Hospital in Würzburg, Germany	In vitro study	PRF loaded with ampicillin/sulbactam	PRF without antibiotics	Antibiotic concentration and antibacterial effects	PRF is highly enriched with ampicillin/sulbactam, and the antibiotic concentration in PRF was comparable to that in the plasma concentration. The IZ of PRF was comparable to the standard ampicillin/sulbactam discs against *H. influenzae*, *S. pneumoniae*, *S. aureus*, and *E. coli.*	PRF is a reliable bio-carrier for systemic applied antibiotics and exhibits a large antimicrobial effect.
Straub A. et al., 2023 [26]	University Hospital in Würzburg, Germany	In vitro study	PRF prepared with three centrifugation protocols (A: 1300 rpm, 8 min; B: 2300 rpm, 12 min; C: 1500 rpm, 14 min) loaded with ampicillin/sulbactam	PRF without antibiotics	Antibiotic concentration and antibacterial effects	A single dose of ampicillin/sulbactam was sufficient to reach high concentrations in PRF in all protocols, which was comparable to the plasma concentration. Protocol B showed the largest inhibition zones against *H. Influenzae*, *S. aureus*, *S. pneumoniae*, *E. coli*, and *P. gingivalis.*	A single dose of ampicillin/sulbactam is sufficient to reach clinically relevant concentrations in PRF.
Straub A., et al., 2023 [27]	University Hospital in Würzburg, Germany	In vitro study	PRF loaded with amoxicillin/clavulanic acid or ampicillin/sulbactam	PRF without antibiotics	Antibacterial effect	A double dose of amoxicillin/clavulanic acid showed higher IZ against *E. coli*, *S. aureus*, *S. pneumoniae*, *H. Influenzae*, and *P. gingivalis* compared to a single dose.	The results demonstrate that oral administration is a suitable route for loading PRF with these drugs. This could expand the scope of PRF application to prevent infections at the surgical site.
Straub A. et al., 2024 [28]	University Hospital in Würzburg, Germany	In vitro study	PRF loaded with clindamycin	PRF without clindamycin	Antibiotic concentration and antibacterial effects	The mean concentration of clindamycin was 0.7 μg/100 mg in PRF, which was significantly lower than in plasma. IZ against *S. aureus*, *S. pneumoniae*, *Streptococcus mitis*, *P. gingivalis*, and *Fusobacterium nucleatum* was significant.	PRF is a suitable bio-carrier for CLI when administered systematically to patients.
Wang S et al., 2021 [29]	Daping Hospital Army Medical University, Chongqing, China	In vitro and in vivo study	PRP in a local antibiotic delivery system (PADS): PRP loaded with vancomycin hydrochloride, clindamycin phosphate, ceftazidime, and thrombin	PRP without vancomycin hydrochloride, clindamycin phosphate, ceftazidime, and thrombin	Antibiotic release kinetics and antibacterial effect	About 60% of the total vancomycin and ceftazidime dose was released within 10 min; then, the release rate gradually decreased. However, 90% of clindamycin was released within 10 min. There was no significant difference in IZD against *S. aureus*, *E. coli*, and *P. aeruginosa* between the test and control groups.	This novel PADS approach might represent a potential therapy for patients who have sustained infected bone defects.

L-PRF, leukocyte- and platelet-rich fibrin; PRF, platelet-rich fibrin; i-PRF, injectable platelet-rich fibrin; MIC, minimal inhibition concentration; T-PRF, titanium platelet-rich fibrin; IZD, inhibition zone diameter; PPD, probing pocket depth, RAL, relative attachment level, RGML, relative gingival margin level, WHI, wound healing index; IZ, inhibition zone.

## Data Availability

No new data were created or analyzed in this study. Data sharing is not applicable to this article.

## Supplementary Materials

The following supporting information can be downloaded at https://www.mdpi.com/article/10.3390/antibiotics13090856/s1, Table S1: excluded studies and reasons; Table S2: summary of the outcomes evaluated in the included studies.
