# Supplementary material for: The Role of Autologous Platelet Concentrates as a Local Antibiotic Delivery System: A Systematic Scoping Review
Source: Antibiotics (Basel). 2024 Sep 6;13(9):856. doi: 10.3390/antibiotics13090856 (PMC11440111; doi:10.3390/antibiotics13090856)
Supplement: Supplementary file 1 [file antibiotics-13-00856-s001.zip › antibiotics-3172523-supplementary.pdf]

Supplementary Table S1: References excluded and reason for the exclusion.

Reference	Exclusion criteria
Anitua E, Nurden P, Prado R, Nurden AT, Padilla S. Autologous fibrin scaffolds: When platelet- and plasma-derived biomolecules meet fibrin. Biomaterials. 2019 Feb;192:440-460.	Narrative Review
Breen A, O'Brien T, Pandit A. Fibrin as a delivery system for therapeutic drugs and biomolecules. Tissue Eng Part B Rev. 2009 Jun;15(2):201-14.	Narrative Review
Drago L, Bortolin M, Vassena C, Romanò CL, Taschieri S, Del Fabbro M. Plasma components and platelet activation are essential for the antimicrobial properties of autologous platelet-rich plasma: an in vitro study. PLoS One. 2014 Sep 18;9(9):e107813.	PRP not involved in antibiotic-delivery
Egle K, Salma I, Dubnika A. From Blood to Regenerative Tissue: How Autologous Platelet-Rich Fibrin Can Be Combined with Other Materials to Ensure Controlled Drug and Growth Factor Release. Int J Mol Sci. 2021 Oct 26;22(21):11553.	Narrative Review
Jakubová, R., Míčková, A., Buzgo, M., Plencner, M., Prosecká, E., Filová, E., Amler, E. Simple drug delivery system based on PRP and PCL nanofibers. European Cells and Materials - Volume 19, Issue 0, pp. 22 - published 2010-01-01.	PRP is not a solely antibiotic-delivery system
Kola SM, Choonara YE, Kumar P, Kondiah PPD, Pillay V. Platelet-inspired therapeutics: current status, limitations, clinical implications, and future potential. Drug Deliv Transl Res. 2021 Feb;11(1):24-48.	Narrative Review
Miron RJ, Zhang Y. Autologous liquid platelet rich fibrin: A novel drug delivery system. Acta Biomater. 2018 Jul 15;75:35-51.	Narrative Review
Nimal TR, Baranwal G, Bavya MC, Biswas R, Jayakumar R. Anti-staphylococcal Activity of Injectable Nano Tigecycline/Chitosan-PRP Composite Hydrogel Using Drosophila melanogaster Model for Infectious Wounds. ACS Appl Mater Interfaces. 2016 Aug 31;8(34):22074-83.	PRP is not a solely antibiotic-delivery system
Rong R, Raza F, Liu Y, Yuan WE, Su J, Qiu M. Blood cell-based drug delivery systems: A biomimetic platform for antibacterial therapy. Eur J Pharm Biopharm. 2022 Aug;177:273-288.	Narrative Review
Tokic T, Duric Z, Gasparovic H. Platelet-Rich Fibrin for Topical Antibiotic Delivery After Sternotomy: Why Aren't We Using It? Ann Thorac Surg. 2023 Dec;116(6):1350-1351.	Correspondence
Wang Q, Qian Z, Liu B, Liu J, Zhang L, Xu J. In vitro and in vivo evaluation of new PRP antibacterial moisturizing dressings for infectious wound repair. J Biomater Sci Polym Ed. 2019 Apr;30(6):462-485.	PRP is not a solely antibiotic-delivery system
Wei S, Xu P, Yao Z, Cui X, Lei X, Li L, Dong Y, Zhu W, Guo R, Cheng B. A composite hydrogel with co-delivery of antimicrobial peptides and platelet-rich plasma to enhance healing of infected wounds in diabetes. Acta Biomater. 2021 Apr 1;124:205-218.	PRP is not a solely antibiotic-delivery system

Yang SC, Lin CF, Alshetaili A, Aljuffali IA, Chien MY, Fang JY. Combining the dual antibacterial and regenerative activities of platelet-rich plasma with β-lactams to mitigate MRSA-infected skin wounds. Biomed Pharmacother. 2023 Se\p;165:115017.	PRP not used as carrier but in combination with antibiotics

Table S2. summary of the outcomes evaluated in the included studies.

Study	Antibiotic(s)	Method/Type of APC	Key Findings
**Antibiotic loading capacity of loaded APC**			
**Bennardo et al. 17**	Gentamicin, Linezolid	L-Tubes	Successful trapping in PRF; analyzed release over time.
**Dubnika 18**	Vancomycin	i-PRF	Without carrier, no controlled loading; used liposomes and microparticles.
**Ercan et al. 20**	Doxycycline	T-PRF	Loaded approx. sevenfold more Doxy compared to collagen (281 ± 43 mg/g vs 47 ± 4 mg/g).
**Straub 2022 25**	Ampicillin/Sulbactam	PRF	PRF enriched with antibiotics; concentrations comparable to plasma.
**Straub 2023 26**	Ampicillin/Sulbactam	PRF	High concentration reached with all preparation protocols; comparable to plasma concentration.
**Straub et al. 2024 28**	Clindamycin	PRF	Mean concentration in PRF significantly lower than in plasma.
**Release kinetics of antibiotic**			
**Bennardo et al. 17**	Gentamicin, Linezolid	PRF	Significant impact on release over time ($p<0.001$).
**Dubnika 18**	Vancomycin	PRF	No controlled delivery without a carrier system.
**Egle et al. 19**	Clindamycin Phosphate	PRF	Burst release in first hour; no significant difference between donor samples.
**Ercan et al. 20**	Doxycycline	T-PRF, Collagen	25% release from T-PRF vs 12% from collagen in 72 hours.
**Knafl et al. 22**	Amikacin, Teicoplanin, Polyhexanide	PRF	Significant release over 24 hours and beyond for some antibiotics.
**Rafiee et al. 24**	Metronidazole, Ciprofloxacin, Minocycline	i-PRF	Burst release within 24 hours, sustained up to 14 days.
**Wang et al. 29**	Vancomycin, Ceftazidime	PRP	60% release within 10 min, gradual decrease; 90% clindamycin release in 10 min.
**Antibacterial effects of loaded APCs**			
**Bennardo et al. 17**	Gentamicin, Linezolid	PRF	Significant antibacterial activity against various strains.
**Dubnika 18**	Vancomycin	PRF	Effective against S. aureus for 48h; rapid drop after 24h.
**Egle et al. 19**	Clindamycin Phosphate	PRF	Decreased bactericidal concentration values against S. aureus, S. epidermidis.
**Ercan et al. 20**	Doxycycline	T-PRF, Collagen	Larger inhibition zones for T-PRF/Dox compared to Collagen/Dox.
**Knafl et al. 22**	Teicoplanin, Amikacin	PRF	Antimicrobial effects verified for almost a week.

**Polak 23**	Metronidazole, Clindamycin, Penicillin	PRF	Significant antibacterial activity with antibiotics; minor without.
**Rafiee et al. 24**	Ciprofloxacin, Metronidazole, Minocycline	i-PRF	Highest activity against A. naeslundii; significant reduction of live bacteria.
**Straub et al. 25**	Ampicillin/Sulbactam	PRF	Comparable inhibition zones to standard discs; no IZ without antibiotics.
**Straub 2023 26**	Ampicillin/Sulbactam	PRF	Protocol B resulted in the largest inhibition zones.
**Straub 2023 27**	Amoxicillin/Clavulanic Acid, Ampicillin/Sulbactam	PRF	Significant differences in IZ between parenteral and oral, and between doses.
**Straub et al. 2024 28**	Clindamycin	PRF	Effective antimicrobial effects against multiple bacteria.
**Wang et al. 29**	Vancomycin, Ceftazidime	PRP	Significant zones of inhibition, similar to antibiotics alone.
**Antibiotics’ impact on APCs structure and viceversa**			
**Bennardo et al. 17**	Vancomycin	PRF	Interfered with PRF formation.
**Egle et al. 19**	Clindamycin Phosphate	PRF	Structural changes in CLP towards a more active form.
**Ercan et al. 20**	Doxycycline	T-PRF	Strengthened the structure of T-PRF.
**Polak et al. 23**	Various Antibiotics	PRF	Significant changes in physical properties with higher volumes.
**Wang et al. 29**	Vancomycin, Ceftazidime	PRP	Reduced growth factor concentration and disrupted structure at higher dosages.
